# CAFFEINE CONSUMPTION AS A POTENTIAL RISK FACTOR OF OSTEOPOROSIS DEVELOPMENT AMONG NIGHT SHIFT WORKERS: EPIDEMIOLOGICAL EVIDENCES AND HYPOTHESIS

**DOI:** 10.13075/ijomeh.1896.02646

**Published:** 2025

**Authors:** Agnieszka Bukowska-Damska, Joanna Jurewicz, Ewa Jabłońska

**Affiliations:** 1 Medical University of Lodz, Department of Physiology, Pathophysiology and Clinical Immunology, Łódź, Poland; 2 Nofer Institute of Occupational Medicine, Department of Chemical Safety, Łódź, Poland

**Keywords:** circadian rhythm, night shift work, osteoporosis, bone mineral density, fracture risk, caffeine consumption

## Abstract

Night workers have demonstrated an increased risk of bone fracture. The mechanisms underlying the observed bone changes among night workers remain unclear. They have been attributed to hormonal changes resulting from exposure to light during nighttime, sleep restrictions and disturbances in expression of circadian rhythms genes. An additional factor that may contribute to increased bone loss among night workers is the consumption of caffeinated products. The aim of the work was to review the epidemiological evidence on the association between caffeine consumption and bone density or fracture risk and to sum up the current knowledge on the association between night shift work and osteoporosis among workers. A search of the literature was conducted in order to identify proper studies using PubMed, Scopus, Elsevier, and Springer databases. A total of 31 articles were identified. The articles were divided to 2 groups of papers assessing the bone fracture risk and osteoporosis among caffeinated beverages drinkers (24 studies) and assessing bone strength in night shifts workers (7 studies). Findings from studies assessing the relationship between caffeine consumption and bone strength appear inconsistent. However, the results of the some presented studies highlight that high caffeine intake increases bone loss. Thus development of osteoporosis among night workers exposed to light during nighttime might be accelerated by high caffeine consumption. No epidemiological study has examined the effect of caffeine intake on the bone fracture risk among night shifts workers yet. There is a great need to better understand the etiology of osteoporosis among workers.

## Highlights

- Night shift workers have demonstrated an increased risk of bone fracture.
- High caffeine intake might increase bone loss.
- Development of osteoporosis in night workers may be accelerated by caffeine intake.

## INTRODUCTION

Osteoporosis is a disease associated with a decreased bone mass leading to an increased risk of low-energy fractures [1]. Osteoporotic fractures the most often occur hip, forearm or vertebrae [1,2]. In the Europe, the number of osteoporosis sufferers is estimated to be nearly 5.5 million men and 22 million women >50 years old. Osteoporosis is a common cause of premature disability or death of elderly people [2,3]. The peak bone mass is reached in 20–30 years of age, following which the level slowly falls [1]. In women, the highest intensity of bone resorption is observed in the perimenopausal period [1,2]. Bone structure is known to be influenced by age, genetic predisposition, ethnic differences, previous fractures, certain diseases or medication; in addition, female sex, lifestyle, and hormone disruptions are also believed to increase the risk [1,4,5]. Many hormones are involved in the pathogenesis of osteoporosis, e.g., estrogen, cortisol or parathormone. The effect of estrogen deficiency after menopause is particularly important, because it promotes osteoclastic bone resorption [1]. In contrast, melatonin has been found to stimulate osteoblast differentiation as well as inhibit bone loss. It also increases synthesis of bone matrix proteins including type I collagen and the activity of bone alkaline phosphatase providing to bone tissue mineralization [3,6–8].

The main source of melatonin in human body is the pineal gland secreting the hormone according with circadian rhythm, with its concentration in blood peaking at night [9]. Circadian activity of all tissues is synchronized by melatonin, including the daily rhythm of bone tissue metabolism, as indicated by *inter alia* diurnal fluctuations of bone turnover markers [9,10–12]. The most important factor limiting melatonin secretion is exposure to light at night. Moreover melatonin secretion is decreased in elderly people and postmenopausal women [9]. Indeed, epidemiological studies have found melatonin secretion to be lower among night shift workers [13–17] and such changes may contribute to progression of osteoporosis, especially among postmenopausal female employees. This was confirmed in 6 of the 7 epidemiological studies on the relationship between night work and osteoporosis development performed to date [18–24].

Night shift workers have demonstrated an increased risk of bone fracture [19,22], decreased bone mineral density [20,23] and increased bone turnover markers [18,21]. However, these surveys were carried out in Asia and America [19,20,22–24] and only 2 have been conducted in European countries [18,21]. Ethnic differences, working conditions and different diet might also be important on observed results.

The mechanisms underlying the observed bone alteration in night workers remain unclear. They have been attributed to hormonal changes resulting from exposure to light during nighttime, sleep restrictions and disturbances in expression of circadian rhythms genes [25]. An additional mechanism leading to increased bone loss by night workers may be frequent consumption of coffee and caffeinated products. Caffeine is an alkaloid that can not only enhance elimination of calcium with urine and inhibit calcium absorption from the gastrointestinal tract, but can also affect biological processes in bone cells [11,26].

Experimental studies have shown that caffeine blocks the repair of damaged DNA and disrupts the cell cycle checkpoints; it also reduces the activity of cyclic adenosine monophosphate (cAMP) phosphodiesterase, leading to inhibition of cAMP hydrolysis: one of the secondary cell messengers involved in maintaining calcium homeostasis in the cellular response to parathyroid hormone (PTH) [27,28].

Parathyroid hormone is synthesized and secreted by the parathyroid glands in response to a decrease in blood calcium (Ca^2+^) concentrations. The physiological activation of the PTH-1 membrane receptor by PTH, through the activation of the G protein, leads to an increase in the synthesis of cAMP by adenylate cyclase. As caffeine is able to inhibit cAMP phosphodiesterase activity, and thus cAMP hydrolysis, its presence may prolong cAMP-dependent protein kinase A (PKA) activity and enhance the cellular response to PTH [1,28].

Osteoblasts are bone-forming cells; they present PTH and vitamin D receptors, together with an ectoenzyme-alkaline phosphatase on their surface, and synthesize the bone matrix proteins. They are known to play a role in regulating bone resorption by activating osteoclasts [1]. The osteoblast cell membrane also houses the receptor activator of nuclear factor kappa-B ligand (RANKL), involved in activation of osteoclasts through the receptor activator of nuclear factor kappa-B (RANK) located on the osteoclasts. The effect of RANKL on osteoclastogenesis is stimulated by activation of the PTH receptor [1]. Thus caffeine may potentially prolong the cellular response to PTH, ultimately resulting in increased bone resorption via intensified osteoclast activity.

**Figure 1.**
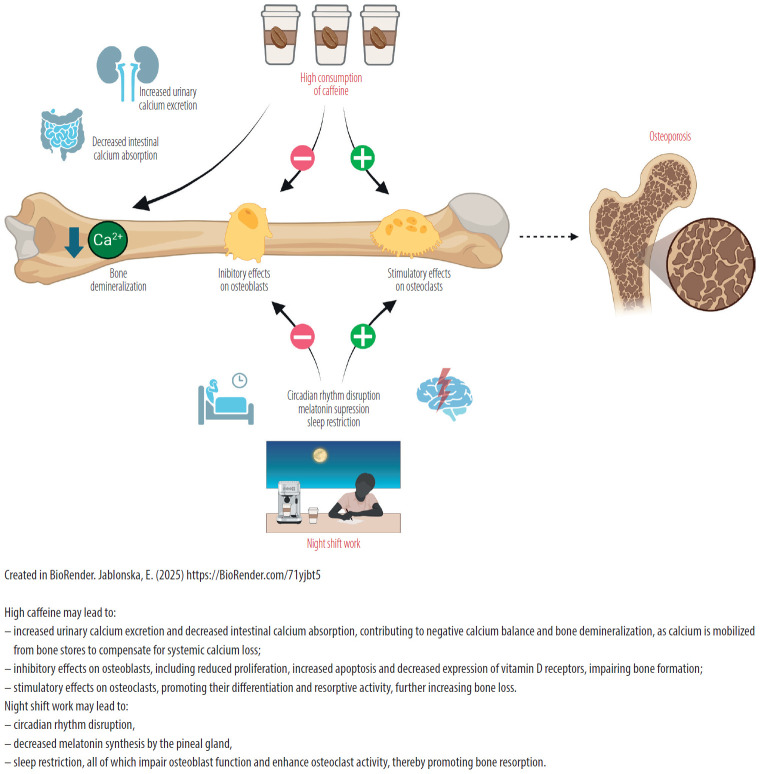
Proposed mechanisms linking high caffeine intake and night shift work with impaired bone metabolism and osteoporosis risk

The results of some experimental studies on human osteoblast cell lines suggest that caffeine inhibits the proliferation and bone formation properties of osteoblasts, induces cell apoptosis and decreases the expression of the vitamin D receptor [29,30]. However, caffeine was not observed to have any effect on the activity of alkaline phosphatase in human osteoblastic cells [30]. It has been also demonstrated that caffeine enhances osteoclast differentiation and the maturation of murine preosteoclast cells [31]. More recent experimental data indicated that the skeletal effects of caffeine are dose dependent and bidirectional [32]. While moderate concentrations inhibited osteoclast differentiation and promoted osteoblast activity, high doses stimulated osteoclastogenesis and suppressed osteoblast function. These effects were modulated mainly via serine–threonine kinase Akt (AKT), nuclear factor kappa-light-chain-enhancer of activated B cells (NF-κB), and mitogen-activated protein kinase (MAPK) pathways. In addition, in an ovariectomized mouse model, moderate caffeine dose (corresponding to approx. 400 mg in humans) inhibited bone loss via decreased osteoclast activity was observed, whereas high dose (corresponding to approx. 800 mg in humans) disrupted bone-forming process and promoted bone mass reduction [32]. Mechanisms by which caffeine may alter bones metabolism is presented in Figure 1. Thus frequent and long-term consumption of caffeine could hypothetically accelerate bone loss, especially in women after menopause with disturbed circadian rhythm, leading to osteoporosis development.

The purpose of this work was to review the epidemiological evidences on the relationship between caffeine consumption and bone density or fracture risk and to sum up the current knowledge on the association between work at night and osteoporosis among employees. This dual focus allows for an integrated view of lifestyle and occupational risk factors contributing to osteoporosis.

## METHODS

The PubMed, Scopus, Science direct, Web of Science, Springer link, and Google Scholar databases were searched to identify relevant epidemiological publications in English, using the terms: “caffeine consumption and osteoporosis,” “caffeine consumption and bone fracture risk,” “caffeine consumption and bone mineral density,” “caffeine and bone turnover markers” as well as “night shift work and bone fracture risk,” “night shift work and bone mineral density” and “night shift work and bone turnover markers.” Additional studies were identified based on references from relevant publications and on citation reports. The review focused on human studies published in peer-reviewed English journals until March 2025. All authors were involved in papers searching. A total of 31 articles were identified. Each paper was evaluated for eligibility. The articles were divided to 2 groups of papers assessing the bone fracture risk and osteoporosis among caffeinated beverages drinkers (24 studies) and assessing bone strength in night shifts workers (7 studies). Animal research, *in vitro* studies, abstracts, notes, review papers, oral presentations, case reports and non-English publications were not considered.

## RESULTS

### Caffeine consumption and bone fracture risk – epidemiological data

A total of 24 epidemiological studies have examined the association between caffeine consumption and bone strength, and have yielded mixed results [33–56]. Among them 8 studies have noted an association between caffeine intake and development of osteoporosis [33,37,39,40,43,45,48,50], 9 studies reported no association [35,39,41,42,44,46,49,51,53] whereas 7 studies indicate for protective impact of caffeine consumption on bone strength [34,36,47,52,54–56]. Summary statement of these studies is presented in the Table 1.

**Table 1.** Summary of epidemiological studies on caffeine consumption and bone fracture risk published in peer-reviewed English journals until March 2025

Country	Population or sample size and age	Study type	Studied outcome and assessment	Caffeine source and assessment	Observed effect
USA [43]	34 703 postmenopausal women, age: 55–69 years	prospective cohort (6.5 years follow-up)	wrist, upper arm, forearm, hip and vertebral fractures reported via questionnaire	coffee, tea and cola reported via questionnaire	– mixed results: correlation between high caffeine consumption and increased risk of wrist fracture (RR for the highest vs. the lowest caffeine intake quintiles: 1.37, 95% CI: 1.11–1.69) – upper arm fracture risk negatively associated with high caffeine intake (RR = 0.67, 95% CI: 0.48–0.94) – no association between caffeine use and hip, vertebral or forearm fractures
Norway [48]	19 752 females, 20 035 males, age: 35–49 years	prospective cohort (11.4 years follow-up)	hip fracture confirmed by review of the individual medical records	coffee reported via questionnaire	– negative in women: increased risk of fracture (RR = 1.85, 95% CI: 1.07–3.17) in those who drank ≥9 cups of coffee per day – no significant association in men
Sweden [39]	31 527 women, age: 40–76 years	prospective cohort (10.3 years follow-up)	all types of osteoporotic fracture identified by matching the unique personal identification number of the study participants with the local outpatients registers, hospital records and X-ray records	coffee and tea reported via questionnaire	– high caffeine consumption (4 cups of coffee) is associated with modestly increased risk of fractures (HR = 1.20, 95% CI: 1.07–1.35) – tea drinking was not associated with fracture risk
Singapore [37]	63 257 men and women, age: 45–74 years	prospective cohort (16.7 years follow-up)	hip fracture identified via record linkage with the hospital discharge database	coffee and tea reported via questionnaire	– high coffee consumption (≥4 cups/day) was associated with higher risk of hip fractures in the total study population (HR = 1.32, 95% CI: 1.07–1.63), and among men (HR = 1.46, 95% CI: 1.01–2.10) and women (HR = 1.33, 95% CI: 1.02–1.72) – no relationship was found with tea consumption in the study population
China [45]	36 740 people, age: 16–101 years	cross-sectional	all types of fracture reported via questionnaire	soft drink, tea, and coffee reported via questionnaire	– drinking soft drinks ≥3 times/week is associated with higher risk of fracture (OR = 1.86, 95% CI: 1.43–2.32) – high tea consumption (≥5 cups/day) was associated with higher risk of fractures (OR = 1.21, 95%CI: 1.09–1.45). – coffee consumption 2 cups/day was associated with increased prevalence of fracture (OR = 1.84, 95% CI: 1.01–3.34)
Japan [50]	249 cases and 498 controls, age: 65–89 years	case-control	hip fracture confirmed by hospitals	coffee reported via questionnaire	– drinking coffee ≥3 cups/day is associated with increased risk of hip fracture (OR = 3.59, 95% CI: 1.46–8.85)
Turkey [33]	80 postmenopausal women, age: 41–65 years	cross-sectional	the lumbar spine and femoral neck bone mineral density estimated by DXA scan	35 different foods and beverages containing caffeine (e.g., coffee, tea, cola, chocolate) reported via questionnaire	– high caffeine consumers obtained similar T-scores to low consumers at the lumbar spine (p = 0.849) – the high caffeine consumers demonstrated significantly lower mean T-scores at the femoral neck compared to low consumers (p = 0.033)
Sweden [40]	359 men and 358 women, age: 72 years (M)	prospective cohort (2 years follow-up)	proximal femur bone mineral density estimated by DXA scan	coffee and tea reported via questionnaire	– men consuming ≥4 cups of coffee/day had 4% lower BMD at the proximal femur (p = 0.04) compared with low or non-consumers of coffee; no such correlation was observed among women – tea consumption was not associated with BMD
China [55]	1817 postmenopausal women, age: 62 years (M)	cross-sectional	calcaneus bone mineral density estimated by QUS	coffee reported via questionnaire	– high frequency of coffee intake was associated with lower prevalence of osteoporosis (OR = 0.54, 95% CI: 0.34–0.84)
China [56]	992 men, age: 30–90 years	cross-sectional	calcaneus bone mineral density estimated by QUS	coffee reported via questionnaire	– the men reporting moderate or high coffee intake had a lower prevalence of osteoporosis development compared to those with low coffee intake (OR = 0.407, 95% CI: 0.174–0.951)
South Korea [36]	4066 postmenopausal women, age: 62 years (M)	cross-sectional	femoral neck and lumbar spine bone mineral density estimated by DXA scan	coffee reported via questionnaire	– women who reported high coffee consumption, had lower odds for osteoporosis compared to those in the reference group (OR = 0.64, 95% CI: 0.43–0.95)
Taiwan [34]	2682 people (1195 men, 706 premenopausal women and 781 postmenopausal women), age: >30 years	prospective cohort (8-years follow-up)	calcaneus bone mineral density estimated by QUS	coffee reported via questionnaire	– high coffee consumption in men was associated with higher T-scores (β = 0.237, p = 0.0067) – coffee drinking was associated with higher T-scores among premenopausal women reporting medium (β = 0.233, p = 0.036) and high coffee consumption (β = 0.234, p = 0.015) – no effect was observed among postmenopausal women
Sweden [52]	7495 men, age: 46–56 years	prospective cohort (30-years follow-up)	hip fracture were verified by X-ray	coffee reported via questionnaire	– coffee consumption is significantly associated with a decreased risk of hip fractures (HR = 0.640, 95% CI: 0.486–0.845) compared to non-drinkers
Finland [47]	1681 women, age: 72 years (M)	prospective cohort (13-years follow-up)	hip fracture confirmed by data from hospital discharge registers	coffee reported via questionnaire	– women who consumed ≥8 cups of coffee/day had significantly lower risk of hip fractures compared to those with a lower daily coffee intake (HR = 0.39, 95% CI: 0.19–0.79)
China [54]	7041 adults (3565 males and 3476 females), age: 20–49 years	cross-sectional	lumbar bone mineral density estimated by DXA scan	coffee reported via questionnaire	– no association between caffeine intake and lumbar bone mineral density in the total population – stratification by age and sex revealed a significant positive correlation between high caffeine intake dose and lumbar BMD in women aged 30–39 years (β = 0.080, p for trend = 0.049), and an inverse association in men aged 40–49 (β = –0.070, p for trend = 0.026) – no significant associations were found for the remaining age groups in either sex
USA [49]	329 women (161 with confirmed first hip fracture and 168 control matched to cases by age group), age >45 years	case-control	hip fracture radiologically confirmed	coffee, tea and soda reported via questionnaire	– no association between consumption of caffeinated beverages and fracture risk
Italy [51]	279 women with hip fracture and 1061 controls, age: 63 years (M)	case-control	hip fracture reported via questionnaire	coffee, decaffeinated coffee, tea and cola reported via questionnaire	– no relationship between frequency and lifelong duration of consumption of coffee, or caffeinated beverages, and the risk of bone fracture
14 centers in 6 countries: France, Greece, Italy, Portugal, Spain and Turkey [44]	730 men with hip fracture and 1132 controls, age: >50 years	case-control	hip fracture identified by the surveillance of hospitals, clinic and nursing homes	coffee reported via questionnaire	– no association between coffee consumption frequency and risk of hip fracture
The Netherlands [53]	16 578 people, age: 25–74 years	prospective cohort (13-years follow-up)	hip fracture confirmed by hospital admission	coffee reported via questionnaire	– no association between coffee consumption frequency and risk of hip fracture
Sweden [42]	42 978 men, age: 45–79 years	prospective cohort (12-years follow-up)	hip fracture data were collected from the National Patient Registry in Sweden	coffee reported via questionnaire	– no significant association between frequency of coffee consumption and risk of hip fracture
USA [46]	6582 men, age: 45–68 years	prospective cohort (31-years follow-up)	hip, spine and forearm fracture reported via questionnaire	coffee reported via questionnaire	– no association between frequency of coffee consumption and prevalence of hip, spine or forearm fracture
South Korea [35]	1761 premenopausal women age: 36 years (M)	cross-sectional	lumbar spine and femoral neck bone mineral density estimated by DXA scan	coffee reported via questionnaire	– no association between coffee consumption frequency and either bone mineral density or risk of osteoporosis
Turkey [38]	2000 postmenopausal women, age: 58 years (M)	cross-sectional	lumbar spine and femoral neck bone mineral density estimated by DXA scan	coffee reported via questionnaire	– no effects of premenopausal coffee consumption on postmenopausal stage bone mineral density was found
Sweden [41]	61 433 women, age: 53 years (M)	prospective cohort (21-years follow-up)	lumbar spine and proximal femur bone mineral density estimated by DXA scan and hip fracture were identified from the Swedish National Patient Registry	coffee reported via questionnaire	– no evidence of a higher rate of hip fracture with increasing coffee consumption – a high coffee intake of ≥4 cups/day was associated with 2–4% lower bone density compared to low coffee consumption, depending on the location; however, the odds ratio for osteoporosis was insignificant

BMD – bone mineral density; DXA – dual-energy X-ray absorptiometry; QUS – quantitative ultrasound.

### Caffeine intake and decreased bone strength

Hansen et al. [43] report a correlation between high caffeine consumption and increased risk of wrist fracture (relative risk [RR] = 1.37, 95% confidence interval [CI]: 1.11–1.69) in 34 703 postmenopausal women followed for 6.5 years during a prospective cohort study. Interestingly upper arm fracture risk was negatively associated with high caffeine intake (RR = 0.67, 95% CI: 0.48–0.94). No association between caffeine use and hip, vertebral or forearm fractures were observed. The multivariate analysis covariates included age, alcohol consumption, tobacco smoking, waist to hip ratio, physical activity level, BMI, estrogen use, calcium intake and calories in the diet. Fractures were also analyzed in relation to dietary sources of caffeine: coffee (cups), tea (cups) and cola (glasses). The age-adjusted relative risk of total fractures was found to be increased, but only for high consumption of cola (≥6 glasses/day) (RR = 1.25, 95% CI: 0.96–1.60, p-trend 0.04) [43].

A prospective study based on dietary data conducted in Norway also found coffee consumption to have an adverse effect on bone strength [48]. The study examined the role of various factors influencing calcium balance, including coffee consumption, in the incidence of hip fracture. Both female (N = 19 752) and male (N = 20 035) participants completed a semi-quantitative dietary questionnaire. The cohort was followed for a mean period of 11.4 years and assessed for hip fracture, defined as neck or trochanteric fracture. It was observed that women who drank ≥9 cups of coffee daily had an increased risk of fracture (RR = 1.85, 95% CI: 1.07–3.17); however, no significant association between coffee intake and hip fracture was noted in men. The multivariate analysis included age at screening, BMI, body height, self-reported physical activity, disability pension, diabetes mellitus, marital status, and smoking habit [48].

A similar observation was obtained in a prospective study in Sweden examining the effect of coffee and tea consumption and estimated total caffeine intake on osteoporotic fracture risk [39]. A cohort of 31 527 women aged 40–76 years at baseline was followed up for 10.3 years. Caffeine intake was assessed based on data collected via a self-administrated food frequency questionnaire. Consumption of 1 cup (150 ml) of coffee corresponded to an intake of 80 mg of caffeine. Women who reported high caffeine consumption (>330 mg/day, equivalent to 4 cups of coffee) had a modestly increased risk of fractures (hazard ratio [HR] = 1.20, 95% CI: 1.07–1.35) compared with those who reported low daily caffeine intake (<200 mg/day). A considerable intake of coffee notably raised the likelihood of fractures (p for trend = 0.002), while consuming tea showed no connection to any risk. Interestingly, among high-level coffee drinkers, i.e., those drinking ≥4 cups per day, a significantly increased fracture risk was observed among women with low calcium intake (<700 mg/day) but not among those with high daily calcium intake (>700 mg/day). The multivariate analysis included age at the study entry, weight, height, vitamin D and A intake, total caloric intake, calcium intake, phosphorus intake, alcohol intake, education level and marital status [39].

Another correlation between high coffee consumption, i.e., ≥ 4 cups daily, and increased risk of hip fracture was also observed in a population-based prospective cohort of 63 257 Chinese men and women between the ages of 45–74 years in Singapore [37]. The study participants completed a semi-quantitative food frequency questionnaire to assess habitual consumption of coffee and tea at the start of the study. The population was followed-up for 16.7 years. High coffee consumption (≥4 cups/day) was significantly associated with higher risk of hip fractures compared to a reference group drinking <1 cup of coffee weekly in the total study population (HR = 1.32, 95% CI: 1.07–1.63), and among men (HR = 1.46, 95% CI: 1.01–2.10) and women (HR = 1.33, 95% CI: 1.02–1.72). A significantly increased risk of hip fracture was also observed among female heavy coffee drinkers, stratified by menopausal status. No relationship was found with tea consumption in the study population. The covariates included in the model were age, sex, BMI, total energy intake, dietary calcium intake, use of vitamins/minerals, smoking status, vegetable-fruit-soy dietary pattern score, dialect group, use of hormone replacement therapy among postmenopausal women, level of education, physical activity, self-reported physician-diagnosed history of diabetes mellitus and stroke [37].

A cross-sectional epidemiological study conducted in China found that consumption of caffeine-containing beverages may have a harmful impact on bone strength [45]. The study included 36 740 participants inhabiting 9 Chinese provinces. Data was acquired on soft drink, tea, and coffee consumption, as well as fracture history and other potential risk factors, via self-administered questionnaires and physical examinations. It was found that consumers drinking soft drinks ≥3 times/week had a higher risk of fracture (OR = 1.86, 95% CI: 1.43–2.32, p < 0.001, p for trend = 0.039) compared with a reference group who did not consume soft drinks. High tea consumption (≥5 cups/day) was also associated with higher risk of fractures (OR = 1.21, 95% CI: 1.09–1.45, p = 0.028, p for trend <0.001). Similarly, study participants who consumed ≥2 cups of coffee daily also had increased prevalence of fracture (OR 1.84, 95% CI: 1.01–3.34, p = 0.045, p for trend = 0.002) compared to non-drinkers. High coffee consumption was significantly associated with increased risk of fractures among both women and men. Interestingly, a significant relationship was found between high consumption of soft drinks or tea and increased risk of fractures in premenopausal women, while no such associations were found among postmenopausal women; however, high coffee consumption was associated with a significantly increased prevalence of fracture among both premenopausal and postmenopausal women. The analysis included age, sex, total energy intake, BMI, dietary calcium intake, alcohol consumption, outdoor activity, smoking status and geographic region as potential confounders [45].

In a case-control study of elderly 249 cases and 498 controls matched by ethnicity, age, sex and residential area, Suzuki et al. also observed that drinking >3 cups of coffee daily is associated with an increased risk of hip fracture (OR = 3.59, 95% CI: 1.46–8.85, p < 0.01) [50].

A negative association was demonstrated between caffeine intake and bone mineral density in a cross-sectional study of 80 Turkish postmenopausal women aged 41–65 years [33]. Caffeine intake was estimated using a food frequency questionnaire. Women who consumed ≥260 mg caffeine/day were classified as high consumers. Bone mineral density (g/cm^2^) was measured by dual-energy X-ray absorptiometry (DXA) at the lumbar spine and femoral neck. The ratio between the bone mineral density of the patient and that of a young adult population of the same sex and ethnicity was calculated as a T-score. According to WHO criteria, a T-score of >–1 was taken as normal, between –1 and –2.5 as osteopenic and <–2.5 as osteoporotic. It was found that among the women, high caffeine consumers obtained similar T-scores to low consumer sat the lumbar spine (p = 0.849); however, the high caffeine consumers demonstrated significantly lower mean T-scores at the femoral neck compared to low consumers (–2.0±0.9 vs. –1.5±0.7, p = 0.033). Moreover, the linear regression model identified a negative correlation between the daily caffeine consumption and the femoral neck T-score in the studied population (R = –0.251, p = 0.025). No similar association was observed for lumbar spine T-score. No multivariate analysis including potential confounding factors was performed [33].

Particularly interesting results were obtained in a study by Hallstrom et al. [40] regarding the relationship between coffee consumption and bone mineral density in men and women, estimated at the proximal femur. The study also examined the role played by genotypes for cytochrome P450 1A2 (*CYP1A2*), associated with caffeine metabolism. Dietary intakes were assessed among 359 men and 358 women (age M = 72 years), based on a 7-day food diary. Bone mineral density for the total proximal femur, femoral neck and trochanteric regions were measured by DXA, and the *CYP1A2* genotypes were determined. It was found that men consuming ≥4 cups of coffee/day had 4% lower bone mineral density at the proximal femur (p = 0.04) compared with low or non-drinkers of coffee. No such correlation was observed among women. Interestingly, among the study participants with high coffee consumption, those with the C/C genotype, associated with rapid metabolism of caffeine, demonstrated lower bone mineral density at the femoral neck (p = 0.01) and at the trochanter region (p = 0.03) than those with T/T or C/T, related to slow caffeine metabolism. Therefore, it is possible that rapid caffeine metabolism may be a potential risk factor for bone loss stimulated by high caffeine consumption [40]. In addition, men and women consuming ≥4 cups of coffee/day with high calcium intake (>1200 mg/day) did not demonstrate higher adjusted mean bone mineral density than those with high coffee consumption and low (<600 mg/day) or intermediate (600–1200 mg/day) calcium intake. Tea consumption was not associated with multivariable-adjusted bone mineral density. All multivariate analyses included age, height, weight, smoking status, physical activity, total caloric intake, intakes of vitamin D, vitamin A, calcium, alcohol and tea [40].

### Caffeine intake and increased bone strength

Some studies suggest that caffeine consumption may prevent osteoporosis. A cross-sectional study of 1817 postmenopausal Chinese women found frequent coffee intake to protect against osteoporosis development [55]. The frequency of coffee intake was assessed by a self-report questionnaire. Bone mineral density was measured using quantitative ultrasound. An univariate analysis demonstrated a significantly lower T-score among women who reported infrequent coffee consumption compared to frequent drinkers (p < 0.001). Similar results were obtained by multivariate linear regression analysis, with a significant positive correlation identified between frequency of coffee consumption and T-score. Moreover multiple variable logistic regression analysis found that high frequency of coffee intake was associated with lower prevalence of osteoporosis (OR = 0.54, 95% CI: 0.34–0.84). The multivariate analysis included age, alcohol intake, education level, smoking, physical activity and medical history [55].

Likewise, a cross-sectional study of 992 Chinese men aged 30–90 years revealed very similar results for the relationship between frequency of coffee consumption and prevalence of osteoporosis [56]. Data on age, education level and lifestyle, including daily frequency of coffee consumption and medical history, were collected using self-report questionnaire. Body weight and height were measured. Bone mineral density was estimated using quantitative ultrasound and a T-score was determined. Univariate linear regression analysis found more frequent coffee intake to be associated with a higher T-score (β = 0.211, p = 0.024). After adjustment for confounding factors, the results of multivariate linear regression analysis remain still significant (β = 0.095, p = 0.032). The men reporting moderate or high coffee intake had a lower prevalence of osteoporosis development compared to those with low coffee intake (OR = 0.407, 95% CI: 0.174–0.951). The multiple regression model included age, alcohol intake, exercise, smoking and medical history as confounding factors [56].

Coffee consumption was found to protect against osteoporosis development in a cross-sectional study of 4066 postmenopausal women from the general South Korean population [36]. The participants completed a questionnaire about coffee consumption. Bone mineral density at femoral neck and lumbar spine were assessed by DXA. After adjusting for confounding factors, the women who reported high coffee consumption, had 36% lower odds for osteoporosis compared to those in the reference group (OR = 0.64, 95% CI: 0.43–0.95). This trend was consistent for osteoporosis of the lumbar spine (p for trend = 0.026) and the femoral neck (p for trend = 0.003). The multivariate analysis included age, BMI, behavioral factors, socioeconomic status factors and hormonal status [36].

A Taiwanese study also suggests that coffee consumption may have beneficial effects on inhibiting osteoporosis development [34]. Briefly, the participants completed a questionnaire on coffee consumption at baseline and after an 8-year follow-up. The group comprised 2682 people, i.e., 1195 men, 706 premenopausal women and 781 postmenopausal women. Coffee intake was categorized into 3 categories (none, medium and high consumption) based on the number of cups consumed per week in both time points, i.e., at start of the study and at follow-up. It was observed that high coffee drinkers had significantly higher T-scores (β = 0.158, p = 0.0038), derived from the osteo-sono assessment index (OSI), i.e., a surrogate of bone mineral density. After separating data by gender, it was determined that moderate and high coffee intake corresponded with increased T-scores; nonetheless, notable findings were mainly observed in men who consumed coffee in large amounts (β = 0.237, p = 0.0067). Regarding menopausal status, coffee consumption was linked to elevated T-scores; significant findings emerged exclusively among premenopausal women who reported medium (β = 0.233, p = 0.036) and high levels of coffee intake (β = 0.234, p = 0.015). Adjustments were made for confounders such as age, education level, BMI, smoking, alcohol dinking, physical activity, vegetarian diet, waist-hip ratio, supplementation with vitamin D and other vitamins or calcium, waist circumference, body fat, diabetes, hypertension, heart diseases, hyperlipidemia and stroke [34].

Coffee consumption was also reported to have a beneficial effect on bone strength in a prospective study on risk factors for hip fracture. The Swedish study followed 7495 men aged 46–56 years for 30 years. Data on occupational class, psychological stress, lifestyle, medical history and anthropometric parameters were collected by questionnaire. Cases of first hip fractures were verified by X-ray. Both the simple and multivariate analyses found coffee consumption to be significantly associated with a decreased risk of hip fractures (HR = 0.640, 95% CI: 0.486–0.845) compared to non-drinkers [52].

A similar result was obtained in a prospective, population-based study of 1681 women (age M = 72 years) followed for 13 years in Finland. Several lifestyle factors including coffee consumption were evaluated as potential causes of hip fracture. Women who reported consuming ≥3 cups of coffee/day had significantly lower risk of hip fractures compared to those with a lower daily coffee intake (HR = 0.39, 95% CI: 0.19–0.79) [47].

A large-scale cross-sectional study of 7041 adults (3565 males and 3476 females) aged 20–49 years by Wang et al. [54] obtained controversial results on the relationship between caffeine consumption and lumbar bone mineral density. Daily caffeine intake was determined based on 2 recall interviews, and bone mineral density was evaluated using DXA method. The researchers did not observe any association between caffeine intake and lumbar bone mineral density in the total population; however, a significant positive correlation was noted among participants aged 30–39 years and 40–49 years, but not in the youngest group (20–29 years). In addition, significant positive correlations between mean caffeine intake and lumbar bone mineral density were noted in the women, but not among the men. Interestingly, stratification by age and sex revealed a significant positive correlation between high caffeine intake dose (the highest quartile) and lumbar bone mineral density in women aged 30–39 years (β = 0.080, p for trend = 0.049), and an inverse association in men aged 40–49 (β = –0.070; p for trend = 0.026). No significant associations were found for the remaining age groups in either sex, and population type did not appear to have any influence [54].

### No association between caffeine intake and bone strength

A case-control study did not identify any association between consumption of caffeinated beverages and risk of hip fracture among 329 women >45 years of age: 161 with confirmed first hip fracture and 168 matched to cases by age group. The analysis included BMI, smoking, education and chronic diseases as covariates [49].

Another case-control study examined the relationship between frequency and lifelong duration of consumption of coffee, or caffeinated beverages, and the risk of fracture among 279 women with hip fracture and 1061 controls [51]. No increased risk of hip fractures was noted among study participants who reported regular coffee consumption compared with non-drinkers. The multivariate analysis also demonstrated a lack of any relationship between frequency of caffeine consumption and risk of fractures, even among women who reported an intake of ≥5 cups of coffee daily; furthermore, long-term coffee consumption for >30 years had no impact on risk of fractures. Similarly, no relationship was found between the consumption of other beverages, such as decaffeinated coffee, tea or cola, and risk of fractures. In the multivariate analysis the results were adjusted for age, BMI, alcohol drinking, education, calcium intake, menopausal status, smoking and estrogen replacement therapy use [51].

The impact of frequency of coffee consumption was also analyzed in a case-control study on risk factors for hip fractures [44]. Hip fracture incidence was determined among 730 men >50 years of age in 14 centers from Portugal, Spain, France, Italy, Greece, and Turkey. Age-stratified controls (N = 1132) were selected from the neighborhood or population registers. Data on occupational work, lifestyle and medical history were collected by questionnaire, as well as anthropometric parameters. Consumption of coffee during childhood, young adulthood and in the recent past was recorded on a 4-point scale: never, sometimes, 1 or 2 cups/day, and ≥3 cups/day. The risk of hip fractures was found to be independent of coffee frequency consumption based on multivariate analysis [44].

Similar results were observed in a longitudinal prospective cohort study on the effect of socioeconomic and lifestyle factors on the risk of hip fractures [53]. The data was obtained by postal questionnaire from 16 578 people aged 25–74 years at baseline. After a 13-year follow-up, it was observed that a high frequency of coffee consumption (≥3 cups/day) at baseline did not influence the risk of hip fracture during follow-up compared to the reference group (non-drinkers). The multivariate analysis included age, sex, paternal occupation, adult occupation, education and income as confounders [53].

Similarly, a large cohort study by Hallström et al. [42] of 42 978 Swedish men, aged 45–79 years old at baseline, did not observe any significant association between frequency of coffee consumption and risk of fractures. Data on coffee consumption were collected by a self-administrated food frequency questionnaire. Data was also recorded on medical history and lifestyle factors. Information regarding the initial fracture at any site, as well as the first hip fracture, was gathered from the Swedish National Patient Registry. Comparing high consumers, i.e., ≥4 cups of coffee/day, to low consumers, i.e., <1 cup/day, the hazard ratio was 0.91 for any type of fracture (95% CI: 0.80–1.02) and 0.89 for hip fracture (95% CI: 0.70–1.14) after a 12-year follow-up. In addition, both crude and adjusted analyses found that even consuming ≥8 cups of coffee/day was not associated with a greater risk of fractures in comparison with men drinking <1 cup/day [42].

Also, no association between frequency of coffee consumption and prevalence of hip, spine or forearm fracture was found in an analysis of 2 prospective studies on risk factors for osteoporosis among 6582 men inhabiting Hawaii [46].

No association between coffee consumption frequency and either bone mineral density (lumbar spine and femoral neck), estimated by DXA, or risk of osteoporosis was found in a Korean cross-sectional study of 1761 premenopausal women. The multivariate logistic regression analysis included age, smoking status, BMI, physical activity, dietary intake of calcium, alcohol consumption, education level and monthly income as confounding factors [35].

A cross-sectional study by Demirbag et al. [38] examined the effects of premenopausal coffee consumption on postmenopausal bone mineral density. The study included 2000 postmenopausal women. Data regarding mean daily coffee consumption (cups per day) in the premenopausal stage, as well as various other data, were collected by questionnaire. Bone mineral density was assessed by DXA. No correlation was found between the amount of coffee consumption and bone density [38].

A longitudinal study of 61 433 women in Sweden also found that high coffee consumption was associated with a moderate reduction in bone density, but that did not translate into an increased risk of fractures [41]. The population-based cohort was followed-up for 21 years. The frequency of coffee consumption was assessed with repeated food frequency questionnaires. In a subcohort of 5022 women, bone density was measured by DXA. Multivariate analysis did not reveal any indication that rising coffee intake is linked to an increased occurrence of fractures. A high coffee intake of ≥4 cups/day was associated with 2–4% lower bone density compared to low coffee consumption, depending on the location. However, the odds ratio for osteoporosis was insignificant [41].

In conclusion, the findings from all above epidemiological studies assessing the relationship between coffee consumption and fracture risk or bone mineral density, appear inconsistent. This difference may be due to the lack of some significant confounding factors in the statistical analysis, such as sex, menopausal status, lifestyle, sex hormone levels, diseases, the use of current medication, as well as the type of coffee, the amount consumed and the method of coffee preparation (e.g., with milk). Moreover, it should be noted that bone turnover is a slow process, and therefore, in addition to investigating the effect of coffee consumption on bone density, researchers should also examine the relationship between coffee intake level and bone turnover marker levels, i.e., bone synthesis such as N-terminal propeptide of type I procollagen (P1NP), and bone destruction such as C-terminal cross-linked telopeptide of type I collagen (CTX). No such relationship has been investigated to date. In addition, no epidemiological study has examined the effect of coffee or caffeinated product consumption on the risk of fractures or bone mineral density among people working on night shifts.

### Osteoporosis among night shift workers – epidemiological data

Seven epidemiological surveys describing the relationship between work at night and bone strength and metabolism were identified [18–24,57]. Summary statement of these studies is presented in the Table 2.

**Table 2.** Summary of epidemiological studies on osteoporosis among night shift workers published in peer-reviewed English journals until March 2025

Country	Population or sample size and age	Study type	Studied outcome and assessment	Observed effect
USA [19]	38 062 postmenopausal female nurses, age: 56 years (M)	prospective cohort (8 years follow-up)	hip and wrist fractures reported via questionnaire	women working on night shifts for ≥20 years have significantly higher risk of fractures than women who have never worked by that system (RR = 1.37, 95% CI: 1.04–1.80)
USA [22]	4408 participants (2351 men and 2057 women), age: 20–80 years	cross-sectional	femoral neck and spine bone mineral density was estimated by DXA scan	shift work was related to an increased prevalence of osteoporosis of the overall population (OR = 2.31, 95% CI: 1.03–5.18) and among women (OR = 4.52, 95% CI: 1.07–19.16) especially among ≥50 years old (OR = 4.60, 95% CI: 1.21–17.54)
Chile [23]	70 postmenopausal nurses (39 women working on rotating night shifts and 31 working only during the day), age: >50 years	cross-sectional	femoral neck and lumbar spine bone mineral density was estimated by DXA scan	women working on night shifts had significantly lower lumbar spine BMD compared to the day workers (L1–L4: M±SD 0.957±0.15 g/cm^2^ vs. 1.104±0.13 g/cm^2^, respectively, p < 0.05), as well as a lower mineral density in both femoral neck bones (right: M±SD 0.936±0.17 g/cm^2^ vs. 1.06±0.12 g/cm^2^, p < 0.05 and left: 0.956±0.19 vs. 1.05±0.12 g/cm^2^, respectively, p < 0.05)
South Korea [20]	3005 individuals (1563 men and 1442 women) working only during the day (2378 people) and working in other than daily system (627 people), age: 36 years (M)	cross-sectional	total femur, femoral neck and lumbar spine bone mineral density was estimated by DXA scan	people working in systems other than daily demonstrated significantly lower mineral density in the total femoral and lumbar spine bones compared to day workers (total femur bone: M±SD 0.948±0.125 g/cm^2^ vs. 0.966±0.125 g/cm^2^, respectively, p = 0.001; lumbar spine: 0.976±0.114 g/cm^2^ vs. 0.988±0.121 g/cm^2^, respectively, p = 0.023)
USA [24]	963 people categorized as daytime workers (369 men and 369 women) and shift workers (101 men and 124 women), age: >50 years	cross-sectional	total femur and femoral neck bone mineral density and bone mineral content was estimated by DXA scan	– male shift workers had significantly higher total femur bone mineral content and femoral neck bone mineral content than regular workers (total femur: 37.33±11.00 g vs. 34.09±10.45 g, respectively, p = 0.01 and femoral neck bone: 4.57±1.07 g vs. 4.29±1.0 g, respectively, p = 0.03) – no association was observed among female subjects
Poland [18,57]	194 female blue-collar workers (90 working on night shifts and 104 working only during the day), age: >40 years	cross-sectional	femoral neck and lumbar spine bone mineral density was estimated by DXA scan and bone turnover markers in blood (P1NP, CTX, FGF-23, osteocalcin, osteopontin, osteoprotegerin) was measured	– no significant association between current or lifetime experience of night shift work and femur or lumbar bone mineral density was observed – significantly higher P1NP, CTX and FGF-23 levels reveal a higher rate of bone turnover among night shifts workers
Italy [21]	82 male subjects: 40 working on night shifts and 42 working only during the day	cross-sectional	bone turnover markers in blood (P1NP, CTX) was measured	serum levels of both CTX and P1NP were found to be higher in night shift workers than in those working only during the day indicating for indicate for higher rate of bone turnover in night shifts workers

BMD – bone mineral density; CTX – C-terminal cross-linked telopeptide of type I collagen; DXA – dual-energy X-ray absorptiometry; FGF-23 – fibroblast growth factor 23; P1NP – N-terminal propeptide of type I procollagen.

Two studies were associated with bone fracture risk among night shift workers [19,22]. According to data gathered from a cohort study including 38 062 postmenopausal nurses over an 8-year follow-up, it was found that the multivariate relative risk of hip and wrist fractures in women who had worked night shifts for a min. 20 years was considerably greater than in those nurses who had never worked night shifts (RR = 1.37, 95% CI: 1.04–1.80). The multivariate analysis included age, physical activity, smoking status, BMI, thiazide diuretic use, hormone replacement therapy use and daily intake of calcium, vitamin D and alcohol [19].

The second epidemiological analysis on the association between night shift work and risk of bone fractures was based on data collected during the National Health and Nutrition Examination Survey (NHANES) cross-sectional study. The analysis comprised 2351 men and 2057 women aged 20–80 years. The multivariate analysis demonstrated that night shift work was related to an increased prevalence of osteoporosis of the overall population (OR = 2.31, 95% CI: 1.03–5.18, p = 0.043) and among women (OR = 4.52, 95% CI: 1.07–19.16, p = 0.041) especially among those aged ≥50 years (OR = 4.60, 95% CI: 1.21–17.54, p = 0.025). The multivariate analysis included *inter alia* age, sex, BMI, blood pressure, ethnicity, nutrient intake, and milk and alcohol consumption [22].

The first study examining the effect of system of work on bone mineral density was conducted on 70 Chilean nurses aged 50 years old [23]. The research team focused on 39 women who have been employed in rotating night shifts for a min. 10 years and 31 other participants who have solely worked daytime hours for ≥10 years. The night shift workers exhibited a notably lower bone mineral density in the lumbar spine when compared to women in the reference group (L1–L4: M±SD 0.957±0.15 g/cm^2^ vs. 1.104±0.13 g/cm^2^, respectively, p < 0.05), and a lower mineral density in both femoral neck bones (right: M±SD 0.936±0.17 g/cm^2^ vs. 1.06±0.12 g/cm^2^, p < 0.05, and left: M±SD 0.956±0.19 g/cm^2^ vs. 1.05±0.12 g/cm^2^, respectively, p < 0.05). Moreover, one quarter of women working on night shifts diagnosed osteoporosis within the lumbar. No similar observation was recorded in daily-active women [23].

The negative effect work during nighttime and bone density was also demonstrated in the South Korean cross-sectional study of 1563 men and 1442 women aged 18–50 years [20]. The study participants were stratified by system of work for day workers (N = 2378) and people working in other than daily systems of work (N = 627), i.e., evening hours, night, and regular and irregular shift work system. The subjects working in systems other than daily demonstrated significantly lower mineral density in the total femoral and lumbar spine bones compared to day workers (total femur bone: M±SD 0.948±0.125 g/cm^2^ vs. 0.966±0.125 g/cm^2^, respectively, p = 0.001; lumbar spine: M±SD 0.976 ± 0.114 g/cm^2^ vs. 0.988 ± 0.121 g/cm^2^, respectively, p = 0.023); however, no differences in femoral neck bone mineral density were found between the groups [20].

However, the NHANES provided results that were inconsistent with these findings [24]. The study involved 470 males and 493 females aged >50 years, who were divided into 2 categories based on their working hours: day shift employees (369 males and 369 females) and night shift employees (101 males and 124 females). The analysis for men and women was conducted separately. The univariate analysis indicated that male shift workers had notably greater total femur and femoral neck mineral content compared to men in the reference group (total femur: M±SD 37.33±11.00 g vs. 34.09±10.45 g, respectively, p = 0.01 and femoral neck: M±SD 4.57±1.07 g vs. 4.29±1.0 g, respectively, p = 0.03). It is also important to highlight that the men engaged in shift work were considerably younger than men in the daytime group (age: M±SD 62.7±9.1 years vs. 65.9±9.7 years, respectively, p < 0.01). No similar observations were described in women [24].

The first epidemiological data in presented topic among Europeans were obtained in a cross-sectional study of female blue-collar workers >40 years of age employed in industrial plants in Poland [57]. The study was carried out among 194 women (90 employees working on night shifts and 104 employees working only during the day). The operating system of work consisted of 3 work shifts: morning, afternoon and night shifts, with 5 consecutive shifts per week followed by a free weekend. When adjusted for important confounding factors, i.e., BMI, ever-used hormone replacement therapy and cigarette smoking, the analysis did not find that current or lifetime experience of night shift work had any significant impact on either femur or lumbar bone mineral density among women stratified by menopausal status [57]. However, bone undergoes constant remodelling via bone resorption followed by bone formation. In the same study population, selected bone turnover markers were measured: P1NP, CTX, osteocalcin, osteopontin, fibroblast growth factor 23 (FGF-23) and osteoprotegerin. A significantly higher level of bone turnover markers were observed in women working on night shifts than among day active employees. This may potentially lead to a future development of osteoporosis even if the bone microarchitecture is not disturbed yet [18].

Similar results obtained in study conducted in Italy including 82 male subjects: 40 working on night shifts and 42 working only during the day. Serum levels of both CTX and P1NP were found to be higher in night shift workers than in those working only during the day. This data indicate for higher rate of bone turnover in night shifts workers and probably may increase risk of development of osteoporosis [21].

In conclusion, 6 of the 7 epidemiological studies indicated a negative effect of night shifts on bone strength. In only 1 study [24] revealed higher bone mineral density among night shift workers than in the reference group. However, it should be noted that the study participants working at night were significantly younger than people included in reference group and that can partially explain observations different from those described in the other studies. Additionally high consumption of caffeine may further accelerate bone turnover among employees leading to premature osteoporosis development.

## CONCLUSIONS

The results of 24 epidemiological studies assessing the impact of coffee consumption on fracture risk or bone mineral density are inconsistent but some studies highlight that high caffeine intake increases bone loss [33,37,39,40,43,45,48,50]. This difference may be due to the estimation of caffeine source and intake assessment. In only 1 study as many as 35 different foods and beverages containing caffeine were included in the analysis as a source of caffeine intake [33]. In other studies the source of caffeine was only coffee [34–36,38,41–44,46–48,50,52–56] or coffee and tea [37,39,40,43,45,49,51]. Moreover frequency of caffeine intake was assessed in all of the 24 studies via questionnaire. Data on coffee consumption such as type of coffee, consumed volume and the method of coffee preparation (e.g., with milk) were not included in the analysis. Thus these are estimated and averaged values of caffeine intake. In addition, no epidemiological study has assessed the relationship between caffeine concentration in blood or caffeine metabolites in urine and bone turnover markers levels *inter alia* P1NP or CTX in blood which may reflect real dose-dependent impact of caffeine on bone turnover process. Finally, none of the described studies included information on the duration of regular coffee intake (exposure expressed in years) or the age at which individuals began consuming caffeine. Both factors can significantly influence long-term effects of caffeine on bone structure. More importantly, these effects may be especially pronounced when caffeine consumption begins during critical periods of bone development such as adolescence or young adulthood.

The review on the association between caffeine consumption and bone strength considered only epidemiological surveys, mostly prospective cohort [34,37,39–43,46–48,52,53] 8 studies were cross-sectional [33,35,36,38,45,54–56] and only 4 were case-control studies [44,49–51].

Bone fracture were mostly confirmed by medical reports or by hospitals [37,39,42,44,47–50,52,53] and in only 4 studies fractures were reported via questionnaire [43,45,46,51]. Bone mineral density was measured mostly at hip and/or lumbar spine [33,35,36,38,40,41,54] using DXA method used commonly in diagnostic properties. However, in 3 studies calcaneus bone mineral density was estimated by quantitative ultrasound [34,55,56].

Another difference in obtained results of studies assessing impact of caffeine intake on bone strength may be due to the lack of some significant confounding factors in the statistical analysis, such as sex, menopausal status, lifestyle factors, sex hormone levels, diseases or the use of current medication. Moreover difference between sample size are also very important. The smallest study included 80 postmenopausal women [33] while the largest study included 63 257 men and women [37]. Finally, study population is very diverse and included both men and women [34,37,40,45,48,50,53,54], only men [42,44,46,52,56] only women [39,41,47,49,51] only postmenopausal women [33,36,38,43,55] or only premenopausal women [35].

To sum up, differences in study design, sample size, population, confounding factors included in the analysis, studied outcome and its assessment as well as data on caffeine source and assessment of intake are the reason of divergent of obtained results.

Osteoporosis is a serious global public health problem. The condition can result in low-energy fractures, which are a primary cause of disability among the elderly and a common cause of death. It is estimated that up to 20% of people who experience an osteoporotic fracture die within the first year due to complications associated with bone fracture [1,2]. Although the current state of knowledge on osteoporosis is extensive, little still is known about the occupational and lifestyle factors potentially affecting bone processes.

Rotating night shift work may be associated with growing number of incidence of osteoporosis [18–23]. The observed relationships have also been attributed to hormonal changes resulting from sleep deprivation and disturbances in expression of circadian rhythm genes associated with nighttime work [19]. Among night workers, bone loss may be exacerbated by frequent consumption of caffeinated products, especially during night shifts when the secretion of melatonin is decreased by exposure to artificial light. The results of both epidemiological and experimental studies indicate that caffeine seems to have more of a negative effect on bone tissue.

It has been estimated that about 90% of adults consume caffeine every day [58]. Moreover, epidemiological studies indicate more frequent or higher coffee consumption among employees working on nighttime shift in contrast to day-active workers [59]. About 20% of people in Europe and the USA work on night shifts [60]. Thus, a large part of the current occupationally active people may suffer due to premature osteoporosis development. The effect of caffeine consumption on bone mass loss among night workers has not been analyzed so far in detail; as such, further studies are warranted. There is a great need to investigate the combined role of lifestyle and occupational factors in the bone remodeling process to prevent osteoporosis: a 21st century civilization disease.

## AUTHOR CONTRIBUTIONS

**Research concept:** Agnieszka Bukowska-Damska, Joanna Jurewicz, Ewa Jabłońska

**Research methodology:** Agnieszka Bukowska-Damska

**Collecting material:** Agnieszka Bukowska-Damska, Ewa Jabłońska

**Interpretation of results:** Agnieszka Bukowska-Damska, Joanna Jurewicz, Ewa Jabłońska

**References:** Agnieszka Bukowska-Damska, Ewa Jabłońska
